# Chances for cure in chronic HBV/HDV coinfection

**DOI:** 10.1186/1471-2334-14-S7-P13

**Published:** 2014-10-15

**Authors:** Gabriela Iliescu, Adriana Bold, Viorel Biciusca, Alexandra Rosu, Daniela Visan, Alice Buteica, Gabriela Tene

**Affiliations:** 1 Emergency Clinical County Hospital Craiova, Romania; 2 University of Medicine and Pharmacy Craiova, Romania; 3 Clinical Hospital Filantropia Craiova, Romania; 4 Clinical Hospital Calafat, Romania; 5 Medical Center SAMA, Craiova, Romania

## Background

Worldwide, chronic infection with HBV and HDV occurs in more than 10 million people with higher rates of developing cirrhosis, hepatic decompensation and hepatocellular carcinoma. Peginterferon (pegIFN) for 48 weeks is one of the current therapies, with poor response rates; prolonging therapy beyond 12 months might be of benefit for the patients.

In order to assess the response to a prolonged pegIFN therapy, we present 2 patients with chronic B/D coinfection, treated with pegIFN alpha 2a, 180 mcg weekly for 96 weeks and monitored, at least, every three months with a complete medical evaluation.

## Case report

Patient 1: Female age 30, diagnosed at age 19 with chronic B/D coinfection; treated the first year with oral azathioprine for 12 weeks, then one week every three months; the second year she started interferon alpha 2a for 48 weeks, achieving biochemical and virologic response. At age 23, she became pregnant, gave birth to a healthy baby girl with no viral infection; after pregnancy, she had an ALT 10 times the upper normal limit, HBV-DNA 1,000,000 IU/mL. For two years, she received support therapy: ursodeoxycholic acid, vitamins, silymarin until we decided to start peginterferon alpha 2a 180 mcg weekly for 48 weeks. Well tolerated, we obtained undetectable HBsAg, HBV-DNA and HDV-RNA in the serum and normal ALT. For a sustained response, we decided to continue this treatment for another 48 weeks. Until present she maintained undetectable HBsAg, HBV-DNA and HDV-RNA; anti HBs < 2.00 (limits 2-10 IU/L), anti-HBe: 0.012 COI (reactive).

Patient 2: Male, 40 years old, diagnosed at age 36 with chronic B/D coinfection; we started standard peginterferon alpha 2a therapy for 48 weeks; with a major side-effect: Hashimoto thyroiditis in month 3, successfully resolved. We continued the treatment, then – also based on a second-opinion from a French hospital – we indicated another 48 weeks of treatment that resulted in sustained biochemical and virologic response with viral eradication, until present.

## Conclusion

A long-term peginterferon therapy could be a chance for cure in patients coinfected with HBV/HDV, with permanent and rigorous monitoring and medical evaluation. This approach needs further studies, for the benefit of patients, considering the higher rates of mortality among them.

